# Human Papillomavirus, Related Diseases, and Vaccination: Knowledge and Awareness Among Health Care Students and Professionals in Nepal

**DOI:** 10.1007/s13187-021-02018-x

**Published:** 2021-05-03

**Authors:** Khawla Suhaila, Amrita Mukherjee, Bipu Maharjan, Amrit Dhakal, Mingma Lama, Anna Junkins, Uddhav Khakurel, Alok Nath Jha, Pauline E. Jolly, Pema Lhaki, Sadeep Shrestha

**Affiliations:** 1 Department of Epidemiology, School of Public Health, University of Alabama At Birmingham, 1665 University Blvd, Birmingham, AL 35294-0022 USA; 2 Nepal Fertility Care Center (NFCC), Lalitpur, Nepal

**Keywords:** HPV knowledge, HPV awareness, HPV cancer, HPV vaccine, Nepal

## Abstract

Human papillomavirus (HPV) is a common sexually transmitted disease worldwide. While burden of HPV-associated cancers and mortality is higher in low-income countries, there is limited data about knowledge of it among health care students and professionals. We assessed awareness and knowledge of HPV, its related diseases, and HPV vaccine among 333 participants, composed of 146 medical students (MSs) and professionals (MPs) and 187 nursing students (NSs) and professionals (NPs) using a 40-question survey between July 2018 and February 2019. Surveys were conducted in English language using both paper and an online version. Most participants reported that they had heard of HPV and cervical cancer. However, 91.76% of MPs and 77.97% of MSs, but only 41.11% of NPs and 36.17% NSs reported knowing that HPV types 16 and 18 caused cervical cancer. Likewise, about two-thirds of MPs and MSs reported having the knowledge that HPV 6 and 11 caused genital warts versus only a little over one-fourth of NPs and NSs. Only 55.91% of NPs and 51.61% of NSs were aware that HPV could cause cancer in both men and women, whereas 42.35% of MPs, 64.41% of MSs, 41.76% of NPs, and 40.66% of NSs were aware that the vaccine could be given to both boys and girls. While medical professionals were relatively more knowledgeable about HPV and related diseases, overall, knowledge about the HPV vaccine was low among all groups. This knowledge gap is concerning and warrants further attention to fight HPV-related public health burden in Nepal.

## Introduction

Human papillomavirus (HPV) spreads through skin-to-skin contact, specifically with sexual contact including vaginal, anal, and oral sex [1, 2]. HPV infection is generally asymptomatic and clears on its own; however, in some individuals, persistence of high-risk HPV types (predominately types 16 and 18) results in pre-cancer lesions and subsequently cancer, while low-risk HPV types (predominately types 6 and 11) result in warts in the ano-genital region and oral cavities [1, 2]. HPV infection prevalence is estimated at 11.7% and accounts for 5.2% of cancer cases [3, 4]. HPV is the second leading infectious cause of cancer globally, causing more than half of all cancers related to infectious diseases in women and 29.5% of all cancers related to infectious diseases worldwide [3, 4]. HPV is known to cause almost all cases of cervical cancer, which is the fourth most common cancer among women and fourth leading cause of all cancer deaths worldwide [3, 4]. However, HPV can also cause a variety of other cancers, including cancer of the anus, oropharynx, oral cavity, penis, vulva, and vagina [1, 5]. An increase in HPV-related anal and head and neck cancers have been reported in the past few years [3, 6]. The prevalence of HPV infection and incidence of HPV-related cervical cancer has decreased over the past decade due to effective screening and vaccination in most high-income countries [3, 7, 8]. However, low-income countries in Asia and Africa continue to have a higher burden of HPV infection and infectious disease-related cancers, and South Asian countries have the highest rates of cervical cancer cases and mortality [4, 7]. Although HPV is suspected as the most common sexually transmitted infection among sexually active couples in Nepal, accurate prevalence and incidence of HPV infection and related cancers are unknown since there is no central cancer registry [9, 10].

The first approved HPV vaccine was in 2006. There are currently three available vaccines, Gardasil (quadrivalent, against HPV types 6, 11, 16, and 18), Cervarix (bivalent, against HPV types 16 and 18), and Gardasil 9 (9-valent, against HPV types 6, 11, 16, 18, 31, 33, 45, 52, and 58) [8, 11]. Current immunization schedule and dosage guidelines vary by age, and recommendations vary by country according to local government and/or World Health Organization (WHO) established guidelines [2, 8, 11]. Prior pilot studies in Nepal have utilized WHO guidelines for vaccine administration, but the program has not been implemented nationally [12]. This variation in dosage typically depends on availability, feasibility, and access to health care facilities for administration and proper storage of the vaccine [13]. HPV vaccination rate is low overall, with approximately 1.4% of the global female population receiving full-course vaccination [14, 15]. High-income countries have a higher proportion of their population vaccinated than low-income countries, and vaccination is often targeted towards adolescent girls [8, 15]. There are disproportionate barriers to full-course vaccination against HPV infection in low-income countries that have a higher burden of cervical cancer [13, 15]. A potential cause for the higher burden of HPV infection and related cancers and lower HPV vaccination rate is a lack of awareness of HPV in general, HPV-related diseases, and HPV vaccine among health care professionals as well as the general population. Efforts to assess and increase awareness of HPV modes of transmission, related diseases and risks, and vaccination are needed worldwide, but especially in low-income countries such as Nepal where incidence of HPV-related cancers are disproportionately higher [3, 10].

There are only a few published studies that assess the knowledge of HPV, its related diseases, and its vaccine in low-income countries, especially among professionals and medical students in health care who are key to prevention, health care delivery, and implementation. Studies of HPV awareness and knowledge have been conducted in the general population of South Asian countries like Bangladesh, India, Indonesia, Malaysia, Thailand, and Sri Lanka; however, most have not comprehensively surveyed both health care professionals and students [16–24]. Previous similar studies in Nepal, including our own studies, also assessed awareness in the general population [24–30]. To our knowledge, there are no published studies on the awareness and knowledge of HPV among health care professionals and students in Nepal. This study was conducted to assess awareness and knowledge of HPV, its related diseases including cancers, and HPV vaccination and related guidelines among medical and nursing professionals and students in Nepal.

## Methods

The study was conducted in conjunction with the Nepal Fertility Care Center (NFCC) in Lalitpur City, Nepal, and the University of Alabama at Birmingham (UAB), School of Public Health, Birmingham, AL, USA, between July 2018 and February 2019 in Nepal. Approval for the study was obtained from the Ethical Review Board at the Nepal Health Research Council and the UAB Institutional Review Board. Informed consent was obtained from all study participants. For participants who completed a paper survey, a physically signed consent form was obtained, and for participants who submitted an online survey, an electronically signed consent was obtained. Inclusion criteria required that the participants be currently enrolled in a medical or nursing program or employed as a health care professional and have English language proficiency. Participation in the study was voluntary and did not include compensation.

### Survey Design

The self-administered questionnaire was designed and developed by investigators at NFCC and UAB, similar to previous studies and national surveys to help ensure validity and understanding [25, 29, 31]. Briefly, the questionnaire included a basic demographics section for age, sex, degree type, indication of professional or student status, year of study (if student), and field specialty (if applicable). The survey consisted of 40 closed-ended questions in multiple-choice format in the English language standardized to Demographic and Health Surveys (DHS) and Nepal Living Standards Surveys (NLSS) format. As in our previous studies, these questions were designed to assess knowledge and awareness of HPV, its related diseases (cancers and warts), cervical cancer screening, HPV vaccination, and related guidelines. Additionally, these survey questions were internally validated by local physicians. Questions that had multiple possible responses were indicated to participants. We used multi-stage random sampling approach to select the participants at local schools and hospitals. There were two methods for delivery of the surveys, a paper and an electronic online version. All personnel conducting the survey were centrally trained to communicate in the community. For the paper surveys, study staff contacted personnel from local institutions with study purpose and survey information to obtain verbal permission to visit and administer surveys in person at select institutions (Paradise Coaching Centers in Ekantakuna and Kupandole, Rise Coaching Center, Medicity Hospital, Bir Hospital, Nidhan Hospital, Yeti Health Scenes, Teaching Hospital in Mahargunj, and Kathmandu Model Hospital). Study staff provided in-person survey information to willing participants at those institutions and obtained written consent from individual participants, as well as monitored participants as they completed the survey. For the online surveys, study staff contacted students and professionals with degrees from health-related programs (MBBS, MD, BN, BSN, and PCLN degrees) using contact lists provided by local organizations (Nepal Medical Council, Doctors Society of Nepal, Nepal Society of Obstetricians and Gynecologists, National Academy of Medical Science, Nepal Medical College and Teaching Hospital Alumni Association, and Lumbini Medical College). Eligibility was verified by phone before sharing survey information and link via email. After explaining the survey study, all participants were consented with physical signature for paper version and electronic agreement for online version.

### Measures and Analysis

The survey responses were entered and combined in a central database and quality control was performed to ensure consistency of responses for both paper and online versions. Any survey question (e.g., variable) with no response, an illegible response, or an invalid response (i.e., multiple responses to single-choice questions) was denoted as missing and omitted from analysis. Any variable with more than 10% missing participant response was not considered for analysis. A univariate descriptive analysis was conducted, reporting frequency (percentage) and proportions within four categories: medical professionals (MPs), medical students (MSs), nursing professionals (NPs), and nursing students (NSs) using chi-square test with an alpha of 0.05. All analyses were performed using SAS 9.4 (Cary, NC).

## Results

### Participants

The survey was completed by 333 participants (153 online and 180 paper surveys). Demographic details of the participants are outlined in Table 1.

**Table 1:** Demographics of survey participants by status

	Medical		Nursing	
	Professionals	Students	Professionals	Students
Participants	87	59	93	94
Median age (yrs)	33	26	30	23
Gender (*N*, %)				
Female	42 (48%)	33 (56%)	90 (97%)	92 (97%)
Male	45 (52%)	26 (44%)	3 (3%)	2 (3%)

### HPV Transmission and Prevention

Overall, a high proportion of participants (100.0% MPs, 98.28% MSs, 96.77% NPs, and 97.87% NSs) had heard about HPV, and of those, 97.65% MPs, 98.28% MSs, 79.57% NPs, and 100.0% NSs learned about HPV in their professional studies (Table 2). In addition, a high proportion of participants (100.0% of MPs, MSs, NSs, and 98.92% of NPs) had heard about cervical cancer, and of those, 87.21% of MPs, 93.22% of MSs, 77.42% NPs, and 91.49% of NSs learned about cervical cancer in their professional studies. The majority of participants (97.67% of MPs, 89.66% of MSs, 92.39% of NPs, and 94.68% of NSs) knew that cervical cancer is preventable.

**Table 2:** Knowledge of HPV and cervical cancer among medical and nursing students and professionals in Nepal

	Medical		Nursing		
	Professional (*N* = 87)	Student (*N* = 59)	Professional (*N* = 93)	Student (*N* = 95)	P*
Have you ever heard about HPV?					.4381
No	0	1 (1.72)	3 (3.23)	2 (2.13)	
Yes	87 (100)	57 (98.28)	90 (96.77)	92 (97.87)	
Did you learn about HPV in your professional studies?					< .0001
No	2 (2.35)	1 (1.72)	19 (20.43)	0	
Yes	83 (97.65)	57 (98.28)	74 (79.57)	93 (100)	
If an individual is HPV positive, will it always result in immediate symptoms?					.2636
No	78 (90.70)	51 (86.44)	74 (83.15)	83 (88.30)	
Yes	7 (8.14)	6 (10.17)	8 (8.99)	4 (4.26)	
I don’t know	1 (1.16)	2 (3.39)	7 (7.87)	7 (7.45)	
There are over 100 types of HPV strains found in humans, do all of them cause cancer?					.0069
No	79 (91.86)	52 (88.14)	69 (75.82)	68 (73.12)	
Yes	4 (4.65)	3 (5.08)	4 (4.40)	6 (6.45)	
I don’t know	3 (3.49)	4 (6.78)	18 (19.78)	19 (20.43)	
Have you ever heard about cervical cancer?					.4614
No	0	0	1 (1.08)	0	
Yes	86 (100)	59 (100)	92 (98.92)	94 (100)	
Was cervical cancer covered in your professional studies curriculum?					.0302
No	11 (12.79)	4 (6.78)	18 (19.35)	6 (6.38)	
Yes	75 (87.21)	55 (93.22)	72 (77.42)	86 (91.49)	
I don’t know/don’t remember	0	0	3 (3.23)	2 (2.13)	
Cervical cancer is preventable					.1257
No	2 (2.33)	2 (3.45)	5 (5.43)	4 (4.27)	
Yes	84 (97.67)	52 (89.66)	85 (92.39)	89 (94.68)	
I don’t know	0	4 (6.90)	2 (2.17)	1 (1.06)	
HPV can cause cancer in					.0001
Men only	1 (1.16)	0	0	0	
Women only	16 (18.60)	8 (13.56)	39 (41.94)	44 (47.31)	
Both men and women	68 (79.07)	50 (84.75)	52 (55.91)	48 (51.61)	
I don’t know	1 (1.16)	1 (1.69)	2 (2.15)	1 (1.08)	

^*^P-value based on Chi-square tests and Fisher’s exact test (count < 5) between the four group of participants (medical professional, medical student, nursing professional, and nursing student)

Knowledge of HPV transmission is presented in Table 3. Although a majority of participants (95.35% of MPs, 98.28% of MSs, 87.10% of NPs, and 87.10% of NSs) were aware that vaginal intercourse could result in HPV transmission, fewer participants knew that HPV could be transmitted through anal intercourse (74.42% of MPs, 67.24% of MSs, 64.52% of NSs, and 39.78% of NSs) and oral intercourse (66.28% of MPs, 58.62% of MSs, 60.22% of NPs, and 31.18% of NSs). Nearly one-fourth of the participants thought that modes of HPV transmission included kissing (saliva exchange) and needle sharing. More than 88% of participants perceived use of condoms as an effective method for preventing HPV transmission; however, many incorrectly perceived vaginal diaphragms as also effective in preventing HPV transmission.

**Table 3:** Knowledge of HPV transmission and prevention among Nepali medical and nursing students and professionals

	Medical		Nursing		
	Professional (*N* = 87)	Student (*N* = 59)	Professional (*N* = 93)	Student (*N* = 95)	P*
How is HPV transmitted?					
Kissing (saliva exchange)	25 (29.07)	15 (25.86)	27 (29.03)	21 (22.58)	.7212
Sharing needles	12 (13.95)	15 (25.86)	25 (26.88)	15 (16.13)	.0807
Vaginal intercourse	82 (95.35)	57 (98.28)	92 (98.92)	81 (87.10)	*.*0017
Anal intercourse	64 (74.42)	39 (67.24)	60 (64.52)	37 (39.78)	< .0001
Non-sexual genital contact	24 (27.91)	12 (20.69)	20 (21.51)	14 (15.05)	.2161
Oral intercourse	57 (66.28)	34 (58.62)	56 (60.22)	29 (31.18)	< .0001
Non-sexual skin to skin contact	15 (17.44)	3 (5.17)	11 (11.83)	9 (9.68)	.1344
I don’t know	0	0	0	1 (1.08)	.4980
What methods can prevent HPV transmission?					
Condoms	77 (89.53)	52 (88.14)	82 (88.17)	76 (80.85)	.3090
Dental dams	6 (6.98)	4 (6.78)	8 (8.60)	2 (2.13)	.2827
Vaginal diaphragms	38 (44.19)	22 (37.29)	34 (36.56)	26 (27.66)	.1459
Birth control pills	1 (1.16)	1 (1.69)	0	1 (1.06)	.7168
Intrauterine device (IUDs)	0	3 (5.08)	2 (2.15)	2 (2.13)	.2227
Vasectomy or laparoscopy	0	4 (6.78)	1 (1.08)	0	.0030
HPV vaccine	77 (89.53)	44 (74.58)	79 (84.95)	74 (78.72)	.0773
Abstinence from sexual activity	53 (61.63)	33 (55.93)	33 (35.48)	35 (37.23)	.0005
Vaginal douching	5 (5.81)	7 (11.86)	7 (7.53)	5 (5.32)	.4452

^*^P-value based on Chi-square tests and Fisher’s exact test (count < 5) between the four group of participants (medical professional, medical student, nursing professional, and nursing student)

### HPV-Related Genital Warts and Cancers

**Fig. 1:**
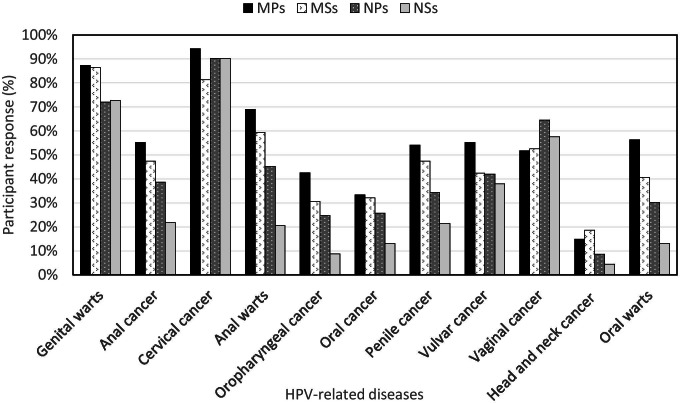
Response rate of knowledge and awareness of different cancers caused by HPV among Nepali medical and nursing students and professionals

As Fig. 1 indicates, most participants knew that an HPV infection can cause genital warts and cervical cancer. However, relatively fewer participants knew that it can also cause anal warts and oral warts. Overall, less than half of the participants knew that HPV can also cause cancers of the anus, oropharynx, oral cavity, penis, and vulva. While over half of the participants were aware that HPV caused cancer of the vagina, less than 20% were aware that HPV also causes cancer of the head and neck.

Most medical participants (91.76% of MPs, 77.97% of MSs) knew that HPV types 16 and 18 account for the majority of cervical cancer cases but nursing participants were less aware (41.11% of NPs and 36.17% NSs). Similarly, a higher proportion of medical participants (69.77% of MPs and 69.49% of MSs) knew that HPV types 6 and 11 account for the majority of warts but less than a third of the nursing participants (29.35% of NPs and 27.78% NSs) were aware of this (Table 2). A higher proportion of medical participants (79.07% MPs and 84.75% MSs) than nursing participants (55.91% NPs and 51.61% NSs) were aware that HPV can cause cancer in both men and women (Table 2).

### HPV Vaccination

Most participants (96.51% of MPs, 89.83% of MSs, 90.22% of NPs, and 89.36% of NSs) knew that there is a vaccine against HPV, but many were not aware that it is available in Nepal (85.88% of MPs, 59.32% of MSs, 68.48% of NPs, and 73.40% of NSs) (Table 4). While most (76.47% of MPs, 52.54% of MSs, 54.95% of NPs, and 56.38% of NSs) knew that the vaccine should be administered before individuals are sexually active, lower proportions (22.35% of MPs, 18.64% of MSs, 41.76% of NPs, and 22.34% of NSs) knew that the vaccine is preventive (should administer before someone is infected with HPV) (Table 4). Overall, most participants (42.35% of MPs, 41.76% of NPs, and 40.66% of NSs) were not aware that the HPV vaccine could be administered to both boys and girls, except for medical students (64.41%) (Table 4). Some participants thought that the HPV vaccine protected against other sexually transmitted infections (STIs) (11.63% of MPs, 18.97% of MSs, 15.22% of NPs, and 28.72% of NSs).

**Table 4:** Knowledge and awareness of HPV vaccine among medical and nursing students and professionals in Nepal

	Medical		Nursing		
	Professional (*N* = 87)	Student (*N* = 59)	Professional (*N* = 93)	Student (*N* = 95)	P*
Is there any vaccine available for HPV?					.2381
No	1 (1.16)	2 (3.39)	2 (2.17)	0	
Yes	83 (96.51)	53 (89.83)	83 (90.22)	84 (89.36)	
I don’t know	2 (2.33)	4 (6.78)	7 (7.61)	10 (10.64)	
Is the HPV vaccine available in Nepal?					.0012
No	6 (7.06)	12 (20.34)	7 (7.61)	5 (5.32)	
Yes	73 (85.88)	35 (59.32)	63 (68.48)	69 (73.40)	
I don’t know	6 (7.06)	12 (20.34)	22 (23.91)	20 (21.28)	
Who can be given the HPV vaccine?					.0136
Boys	0	0	0	0	
Girls	48 (56.47)	18 (30.51)	47 (51.65)	52 (57.14)	
Boys and girls	36 (42.35)	38 (64.41)	38 (41.76)	37 (40.66)	
I don’t know	1 (1.18)	3 (5.08)	6 (6.59)	2 (2.20)	
When do you think HPV vaccine should be given?					
After individuals are sexually active	11 (12.94)	18 (30.51)	27 (29.67)	41 (43.62)	.0002
Before individuals are sexually active	65 (76.47)	31 (52.54)	50 (54.95)	53 (56.38)	.0059
After an individual is HPV positive	2 (2.35)	7 (11.86)	7 (7.69)	8 (8.51)	.1634
Before an individual is HPV positive	19 (22.35)	11 (18.64)	38 (41.76)	21 (22.34)	0028
As children	13 (15.29)	19 (32.20)	20 (21.98)	9 (9.57)	.0035
I don’t know	0	7 (11.86)	3 (3.30)	4 (4.26)	.0062
Do girls/women who have already been vaccinated require cervical cancer screening?					.0901
No	5 (5.81)	7 (12.07)	10 (11.11)	15 (16.13)	
Yes	78 (90.70)	43 (74.14)	70 (77.78)	70 (75.27)	
I don’t know	3 (3.49)	8 (13.79)	10 (11.11)	8 (8.60)	
Does the HPV vaccine protect against other sexually transmitted infections (STIs)?					< .0001
No	76 (88.37)	34 (58.62)	70 (76.09)	58 (61.70)	
Yes	10 (11.63)	11 (18.97)	14 (15.22)	27 (28.72)	
I don’t know	0	13 (22.41)	8 (8.70)	9 (9.57)	

^*^P-value based on Chi-square tests and Fisher’s exact test (count < 5) between the four group of participants (medical professional, medical student, nursing professional, and nursing student)

## Discussion

This study is the first to examine awareness and knowledge of HPV, its related diseases, and vaccine among medical and nursing students and professionals in Nepal. The majority of medical and nursing students and professionals reported awareness of HPV and its relation to cervical cancer development. Compared to both medical and nursing students, knowledge was relatively higher among medical professionals and lower among nursing professionals. Overall, all participants exhibited more general knowledge about HPV and less specific knowledge about related diseases and vaccination. Our study findings exhibit similar trends in knowledge as other published studies where general HPV knowledge was higher than knowledge about HPV-related diseases and HPV vaccine [32–36]. While knowledge that HPV causes cervical and vaginal cancers was relatively high in our study, most participants were not aware of its associations with anal, oral, and head and neck cancers. Head and neck cancer is often associated with tobacco use but knowledge of HPV as a risk factor and its transmission through oral sex is limited. This is corroborated by findings from our study. While the association of HPV and cervical cancer is well established, some health care professionals may not be aware of the associations between other cancers that are currently known to be caused by HPV, such as cancers of the anus and oropharynx, as evidenced by low response rates in our study. Likewise, they may not be as knowledgeable about the effectiveness of the vaccine against other HPV-related cancers. A general lack of awareness regarding the HPV vaccine could be due to the relative novelty of the vaccine and ongoing data reports [37, 38]. Furthermore, vaccination guidelines vary in low-income countries like Nepal and HPV vaccination initiatives are often directed towards girls to prevent cervical cancer [8, 37]. Thus, participants’ response to who can be given the vaccine could be reflective of local vaccination protocols. However, these HPV vaccination protocols potentially contribute to the higher risk of developing HPV-related cancers in men, especially anal and oropharyngeal, as evidenced by the increasing incidence of these cancers [39, 40].

Most previous studies surveyed health care professionals or students in specific fields [18, 34, 35, 41–43]. However, there are limited studies that comprehensively report on general knowledge of HPV infection, screening, vaccination, and related diseases among health care professionals and students. One small study observed that health science students were more aware of HPV (98.8%) than non-health science students (47.5%), but it did not assess awareness among health care professionals, nor did it assess disease outcome-based knowledge [27]. Also, unlike our study, most previous studies focused on HPV in relation to vaccination or cervical cancer [19, 32, 44–46]. Although our study was conducted in and with participants from Nepal, the results of this study could be reflective of other South Asian countries that have a similar health care education curriculum and from which some Nepali health care professionals receive their degree. The generalizability of this study is therefore limited to health care professionals and students in the South Asian region.

Nursing professionals were consistently less aware of HPV-related diseases and vaccination. Although Nepal has accreditation and licensing examination overseen by the Nepal Nursing Council, there is no established requirement for continuing education or training for practicing nursing professionals. Continuing education and training after the licensing exam is essentially voluntary, sporadic, and organized mainly by outside organizations [47]. The lack of a standard continuing education component for professional development could be a significant contributing factor for decreased knowledge and awareness of HPV and associated cancers among nursing professionals in Nepal. Apart from the cervix, HPV infection prevalence is slightly higher in men than women worldwide, and the associated cancers (anal and oropharyngeal) are thus also more common and are increasing in men [3, 40]. Even with vaccination recommendation expanded to all adolescent children (both boys and girls) in countries like the USA, HPV vaccination guidelines and efforts continue to be focused on adolescent girls, and the World Health Organization schedule for HPV vaccination explicitly recommends it for females [8, 40].

This is one of few studies of its kind in a South Asian country and the only one in Nepal. This is significant from multiple perspectives as it is related not only to medical education standards and practice, but also cultural taboos about sexual behavior and health. Discussion of sex in general, specifically oral and anal sex, and sexually transmitted infections is not common. This is especially true in South Asian countries like Nepal. One study reported that 24.5% of its adolescent participants acknowledged engaging in oral sex and 19.5% in anal sex [48]. While these sexual practices are prevalent, difficulty in addressing sexual health is well recognized in Nepal. Even among medical professionals, sex-related topics are often discussed in English or Sanskrit-derived terminology but not in Nepali terms [49]. However, all participants completed the survey in its entirety, which is an indication that these types of questionnaires were well received within our target population.

There are several limitations to be considered in the interpretation of the results from the current study. First, collection of additional participant characteristics would have been beneficial in order to conduct analyses determining demographic and training variables associated with knowledge. For example, inquiry of duration of practice and location of degree/internship of professionals could have been valuable to illuminate additional potential causes of knowledge gaps. Second, there is potential respondent bias with the online version of the survey because there was no time limit nor any monitoring system to prevent use of outside resources (unlike the paper version). Furthermore, response bias between paper and online versions may be present because most paper surveys were taken by students whereas most online surveys were taken by medical professionals. However, in the current study, it was not possible to measure and distinguish if any bias occurred due to survey format (paper/online) or occupation (student/professional).

## Conclusion

Our study found that although health care professionals had general factual knowledge of HPV, more specific knowledge applicable to their medical practice was lacking. Within our study population, medical professionals exhibited the most and nursing professionals exhibited the least knowledge regarding HPV. Findings from our study could be used to advocate for establishing standardized and comprehensive education on HPV, related diseases, and vaccination in the health care education curriculum and continuing education requirements. In general, the lack of knowledge among some of the health care professionals and students is concerning. It may be appropriate to explore if integration of explicit HPV education into the curriculum would be beneficial. Future studies with a larger sample of students and professionals in various health care disciplines in Nepal would provide information that could be helpful in revising the educational curriculum and program at all levels and stages. Additional studies on the knowledge of health care professionals and students in areas with high incidence of HPV infection and related cancers are needed, especially in South Asian countries like Nepal.
